# HSDDD: A Hybrid Scheme for the Detection of Distracted Driving through Fusion of Deep Learning and Handcrafted Features

**DOI:** 10.3390/s22051864

**Published:** 2022-02-26

**Authors:** Monagi H. Alkinani, Wazir Zada Khan, Quratulain Arshad, Mudassar Raza

**Affiliations:** 1 Department of Computer Science and Artificial Intelligence, College of Computer Science and Engineering, University of Jeddah, Jeddah 21959, Saudi Arabia; malkinani@uj.edu.sa; 2 Department of Computer Science, University of Wah, Wah Cantt 47040, Pakistan; 3 Department of Computer Science, Comsats University Islamabad, Wah Campus, Wah Cantt 47040, Pakistan; brightsuccess_12@yahoo.com (Q.A.); mudassarraza@ciitwah.edu.pk (M.R.)

**Keywords:** diver distraction, deep learning, kNN, SVM, HOG, handcrafted features, Alexnet, Inception V3, Resnet50, VGG16

## Abstract

Traditional methods for behavior detection of distracted drivers are not capable of capturing driver behavior features related to complex temporal features. With the goal to improve transportation safety and to reduce fatal accidents on roads, this research article presents a Hybrid Scheme for the Detection of Distracted Driving called HSDDD. This scheme is based on a strategy of aggregating handcrafted and deep CNN features. HSDDD is based on three-tiered architecture. The three tiers are named as Coordination tier, Concatenation tier and Classification tier. We first obtain HOG features by using handcrafted algorithms, and then at the coordination tier, we leverage four deep CNN models including AlexNet, Inception V3, Resnet50 and VGG-16 for extracting DCNN features. DCNN extracted features are fused with HOG extracted features at the Concatenation tier. Then PCA is used as a feature selection technique. PCA takes both the extracted features and removes the redundant and irrelevant information, and it improves the classification performance. After feature fusion and feature selection, the two classifiers, KNN and SVM, at the Classification tier take the selected features and classify the ten classes of distracted driving behaviors. We evaluate our proposed scheme and observe its performance by using the accuracy metrics.

## 1. Introduction

A scrutiny by WHO (World Health Organization) has come up with the statistics of death of 1.3 million people every year due to the accidents on roads, and from the last few years, this figure is becoming larger. Some of them suffer from severe injuries, while others lose their lives. According to the most heartbreaking statistics, the leading cause of death among people aged 5 to 29 was road traffic injury. According to NHSTA, in 2020, traffic crashes of motor vehicles resulted in an estimated death of 38,680 people, whereas in 2019, an estimated 36,096 people died in road accidents. This shows an increase death rate of about 7.2 percent. These road accidents lead to serious damages to both humans as well as their properties, causing substantial amount of economic damage for persons and their families. This will ultimately impose serious problems on society affecting the nations as a whole [1]. Thus, understanding reasons and factors associated with these road accidents is very crucial. Although there are many different elements and origins that cause automobile accidents on roads, distracted driving is found to be an important cause that contributes significantly to fatal vehicle crashes and injuries. In an investigation, it was shown that human operations and errors are the main causes of car collisions, and in the United States, 94% of traffic accidents on roads are the result of distracted driving, which is the most dangerous behavior that can reduce a driver’s reaction speed. According to the report in [2], driver’s multitasking while driving is a leading cause of traffic accidents, conveying that driver distraction is considered to be the cause of most of the traffic collisions. Furthermore, research done by the National Highway Traffic Safety Administration (NHTSA) between 2014 and 2019 has revealed that a distracted motorist is responsible for approximately a quarter of all traffic accidents.

A distraction of a driver is considered to be anything that causes inattention or losing of concentration behind the wheel. Distracted driving occurs when a driver’s attention is diverted from controlling the vehicle while driving. Driving performance of a human driver declines due to his distraction, which ultimately results in vehicle crashes when the vehicle becomes out of control and the vehicle starts drifting outside lane changes or changes speed in an unplanned manner. Distraction can be originated in two ways: first, distraction initiated by the driver when he starts doing activities other than driving, and second, distraction not initiated by the driver but by someone else, and unpredictable actions of something distracts the driver’s attention [3]. Distractions of a human driver can be of different types according to their severity and form. Distraction can be caused by using a mobile phone, taking a call, reading or texting a message, glancing at persons on the roads and events happening outside the vehicle, smoking, drinking or eating [2]. Moreover, using any applications for entertainment or navigation or smart devices including car’s infotainment systems or smart watches and can also divert the driver’s attention while driving. There are six types of human driver distractions, namely manual, visual, cognitive, olfactory, gustatory, and auditory distraction. A driver who is taking his/her hands off the steering wheel is involved in distraction called manual distraction. A driver who is taking his/her eye off the road is distracted visually and is involved in distraction called visual distraction. A driver is involved in cognitive distraction when he is taking his/her mind off of driving by thinking about the things other than driving. A human driver is involved in auditory distraction when he is hearing music or some ringtones which make him less attentive towards safe driving and may obstruct him from driving carefully. A distraction is a kind of olfactory distraction in which smell of something outside or inside the car diverts the attention of a human driver. When a human driver is eating or drinking something, he is distracted by its taste and thus is involved in gustatory distraction. Hence, driving is a process that requires multitasking which involves collective attentional resources form motor responses, spatially working memory and visual perceptions. Usage of cell phones is a kind of distraction that involves an amalgamation of manual, visual, and cognitive distractions, and hence, according to the National Safety Council [4], cell phones are involved in 26% of all car accidents. To avoid potential safety risks, there is a need to monitor, detect and predict early the human driver inattentive behavior, and necessary warnings should be provided to the driver by accurately classifying a distracted driving state and nondistracted driving state. Researchers have attempted to mitigate this problem by leveraging AI for developing driver assistance and alert systems by understanding risky driving behaviors [5,6]. Detection of a distracted human driver can be categorized into two types of measurement strategies, measuring visual behavior of human driver and measuring vehicle related features or vehicle dynamics [7].

The existing traditional machine-learning-based techniques employ manual design for extracting features [8,9]. Thus, these methods are highly subjective and dependent upon expert experience. Moreover, they have poor robustness because transferring of features is not an easy task using these methods. The existing driver distraction detection techniques using CNN models (AlexNet, VGG, ResNet50, Inception-V3, etc.) and other deep networks including RCNN, Faster RCNN, LSTM have promising results with significant performance improvements when researchers employ different variations of CNN models for the identification of postures of a distracted driver. However, there are still some limitations, and in most of the research papers, the authors have selected the best model on the basis of performances of state-of-the-art CNN models by simply evaluating them for the image classification of distracted driving. Thus, there is a lack of well-defined objectives. Another problem with deep learning models is that when researchers make hyperparameters and the network structure design adjustments manually in a complicated way and when done specifically, for distracted driver detection, these processes become a complicated task because it necessitates a significant amount of testing, tweaking and adjustments. Moreover, the deep neural network, which was manually constructed, may not be an appropriate architecture for detecting distraction of a driver. Furthermore, another unresolved problem regarding the usage of these deep networks for the identification of distracted drivers is that in these studies or techniques, detection of a distracted driver is accomplished by analyzing images or videos of the driver’s posture from his upper body; however, time series data such as vehicle dynamic information and physiological signals have not been experimentally evaluated or incorporated in these deep networks for the detection of driver distraction.

The goal of this paper is to detect manual distraction of a human driver when he is not driving safely and is engaged in other activities which can distract him. We present a Hybrid Scheme for the Detection of Distracted Driving called HSDDD. This scheme is based on a strategy of aggregating handcrafted and deep CNN features. The main contributions are as follows:

- The proposed HSDDD is based on three-tiered architecture. The three tiers are named as Coordination tier, Concatenation tier and Classification tier.
- We have aggregated the extracted features from the four pre-trained CNN models as deep automatic features along with the HOG features as classical handcrafted features. This work is an attempt to overcome the limitations of models based on deep learning and gain the benefits of both kinds of feature extraction methods i.e., deep learning-based methods and handcrafted based methods.
- The outcomes are compared with existing Deep-Learning-based techniques.

The remainder of the paper is organized as follows: Section 2 presents the most recent literature concerning the detection of a distracted human driver. Section 3 describes our proposed hybrid scheme for distracted driving detection. Section 4 demonstrates experimental results of our proposed scheme and present a comparison with some of the existing techniques. Section 5 finally presents the conclusion of the paper.

## 2. Recent Literature

Since 2016, researchers have started using CNN Deep Learning models for the detection of distracted drivers [10,11,12,13,14]. C. Streiffer et al. [11] have built an analysis framework called DarNet for distracted driving behavior detection and classification. In this framework, data is collected in a unified manner, and then aggregation of convolution and recurrent neural network is utilized for analyzing the images of distracted driving. Moreover, The DarNet has learned to detect six classes of distracted driving behaviors by leveraging an IMU sensor data, resulting in an increased accuracy. H. M. Eraqi et al. [14] have introduced a CNN-based technique in which a learnable weighted ensemble is used. In order to obtain the input in the form of RGB images of a distracted driver, a dashboard with a mounted camera is utilized. The network is initialized with a set containing generic features which in turn enable high precision in recognizing a wide range of objects. Different postures of a distracted driver other than ImageNet classes are recognized by the latent fully connected layers including 10 driving distraction postures. A. Koesdwiady et al. [15] have introduced an end-to-end CNN-based solution for identifying distracted drivers. In this scheme, the authors have used VGG19 for feature extraction resulting in the achievement of 95% accuracy as the highest in the testing phase. M. Leekha et al. [16] have presented a CNN-based architecture which is robust and simple and involves foreground extraction. In their design, 0.5 M parameters are used which are fewer than the existing models and ConvNet model consists of 3 × 3 filters. In the successive layers, these filters are progressively increasing, finally achieving improved accuracy and the false positive rate is lower. J. M. Celaya-Padilla et al. [17] have presented a method which is adapted ubiquitously for the detection of drivers who are distracted by using their cellphones. For this research study, the authors have used a wide-angle camera mounted on a ceiling which is integrated with a deep learning CNN. Training of Inception V3 network is performed for the detection of images showing the drivers who are driving and also texting on their cell phones.

In the literature, the distracted driver recognition methods can be mainly categorized into two major types: methods based on hand-crafted feature extraction and methods based on Deep CNN feature extraction. Previously, some handcrafted feature-based methods [8,18,19] were used for the detection of distracted drivers. In these techniques, scale-invariant feature transform (SIFT) and Histogram of Oriented Gradient (HOG) were used for extracting the features, and then a classifier such as Support Vector Machine (SVM) was used to classify whether the driver is distracted or not, but these techniques achieved the accuracy of 40% and less than 40% on the Kaggle State Farm Dataset. Different Machine Learning (ML)-based techniques including HCRF (Hidden Conditional Random Field), HMM (Hidden Markov Model) and SVM (Support Vector Machine) were also used for driver distraction [20,21,22]. More recently, researchers have started using models based on Deep Learning to detect and identify distracted drivers.

J. M. Mase et al. [23] have proposed a methodology for detecting the posture of a distracted driver in which the spectral-spatio features of the images are captured using CNNs and stacked BiLSTM Network (Bidirectional Long Short-Term Memory). In the proposed idea, there are two stages. In the first stage, the features related to spatial posture are automatically learned by leveraging CNNs that are already pre-trained. In the second stage, BiLSTMs architecture is utilized for the extraction of spectral features from pre-trained CNNs which are among the stacked feature maps. Evaluation of the proposed methodology is performed on the AUC dataset showing an accuracy of classification as 92.7%.

M. Wu et al. [24] have proposed a novel multifeature fusion network for the detection of image based distracted driving which is based on pose estimation. In the proposed method, hands are first detected by utilizing the information about the postures of human body. Global features and the features related to pose and hand are extracted. After feature extraction, the fusion of hand and pose features and global features is performed by combining the weights of probability vectors, and features maps are concatenated. The evaluation of the proposed method is performed by observing its performance on two datasets; first, experiments are performed on the public AUC Distracted Driver dataset and then on their own dataset called SZ Bus Driver dataset. Achieved accuracies are 95.75%, 96.2% and 90.3% on SZ Bus Driver dataset, AUC V1 and AUC V2 dataset, respectively.

C. H. Jin et al. [25] have proposed a deep-learning-based scheme for driver behavior detection by identifying the cell phone usage and giving an alert. This technique has followed two stages: the first stage is model training and the second stage is practical testing. After creating a multi-angle camera configuration, two separate CNNs are trained using a self-defined data set in the model training stage and the number of the convolution kernels, and their size are both optimized. Due to this optimization, hands and cell phones can be recognized in an efficient manner in real time. The second stage of testing has employed skin color detection and dynamic region extraction so that the accuracy of target recognition can be improved. Then, the human hands and the cell phones are detected by leveraging the trained CNNs. The interaction between the cell phone and human hand is observed, and the distance is calculated, which is then used as a basis for notifying the warnings. The results of experiments have shown the accuracy of 95.7% with a running time of 144 fps in detecting cell phone usage.

C. Huang et al. [26] have presented HCF, a hybrid CNN framework for detecting distracted behaviors of drivers. HCF is based on three modules. The first module is a cooperative CNN module in which multiscale behavior features are extracted in parallel by using a combination of three pretrained models, Inception V3, ResNet50, and Xception. The second module is a feature concatenation module in which the independent features extracted by the pre-trained models are concatenated to obtain comprehensive information and a set of fusion vectors of one dimensional is formed by deeply fusing the extracted features. In the third module, there is a feature classification module in which HCF has fully connected layers which are trained to detect the movements of human hands so that the distracted and nondistracted driving behaviors can be classified. HCF has neurons in its fully connected layer which are responsible for capturing important elements of the feature vectors that are fused together and that are used as a basis for classification of different distracted driving behaviors. In order to avoid an overfitting problem in the data during training HCF, an improved dropout algorithm is applied. For evaluation, the class activation mapping (CAM) technique is used so that the ten classes of behaviors of distracted drivers in the feature area can be highlighted. The results of experiments performed have shown that HCF has achieved the accuracy of 96.74% in classifying and detecting the behaviors of distracted drivers.

A. Gumaei et al. [27] have proposed a camera based deep learning framework for real time identification of driver’s distraction by leveraging cloud computing and edge computing technologies. In this paper, the authors have selected two models including the CDCNN model, which stands for custom deep convolutional neural network, and fine-tuned VGG16 model, which stands for visual geometry group-16, and have also investigated their efficacy. The authors have proposed a framework with three modules; distraction detection module, training module and the analyzing module. In the vehicle environment, the edge devices have the first module, i.e., the distraction detection module. The trained detector has taken the input in the form of frames of distracted behavior of a driver by connecting to a fixed camera device. When distracted driving behavior is identified by the trained detector, an alert to the driver is sent by using the telecommunication-based internet connection. An image of driver distraction is also released by the detection module to the cloud computing side that contains a text message and label displaying the distraction. The cloud environment has the second module i.e., the training module which uses the initial dataset to produce detectors by learning through a deep learning approach. The monitoring environment (administrator side) has the third module, i.e., the analyzing module which is connected with a telecommunication network and is used to send alert messages by analyzing the driver behavior. Reports are generated by the analyzing module which contains the number and types of distractions with the name of the driver so that driver behavior can be monitored easily. The evaluation of the two models is performed by doing experiments on the dataset of driver distraction that is large scale and is publicly available. When 10% holdout test set is used, accuracy rate for the first model is 99.64%, and it is 99.73% for the second model. When 30% holdout test set is used, the first model has achieved the accuracy of 99.36%, whereas the second model has achieved the accuracy of 99.57%.

W. Zhu et al. [28] have proposed a framework based on the multi-layer perception (MLP) and long short-term memory (LSTM) model. The proposed methodology contains four parts: data processing, LSTM neural network for incident clearance prediction and MLP network for incident clearance prediction. To predict the short-term traffic incident duration, two kinds of variables, traffic-related factors and traffic flow data, are applied as inputs of the framework. In the first part, traffic flow data and incident-related factors are first being processed. Data processing has two parts, slide the time window to obtain more samples and data normalization. In the normalization part, the raw data is normalized to ensure the reliability of the results and the equal contribution of each factor. The second part is an LSTM neural network for incident clearance prediction in which an LSTM neural network consists of one input layer, one hidden layer and one output layer. The third part is an MLP network for incident clearance prediction which consists of one input layer, one or more hidden layers and one output layer. This study selected the traffic incident data and traffic flow parameters in Shanghai Zhonghuan Expressway as a case study. A total number of 4041 incident records were originally collected, covering the period from 1 April 2017 to 7 October 2017. This paper has provided the ROC curve, KS curve and confusion matrix of the proposed model with optimal parameter combination.

D. Tran et al. [29] have proposed a driver distraction detection system in which camera is used to identify various types of distractions by observing the driver. To create realistic driving experiences and to validate the distraction detection algorithm, an assisted-driving testbed is developed and experiments were performed. The assisted-driving testbed has four main components: (i) a driving simulator which simulates the driving environment; (ii) a software tool that helps users design road maps for the driving simulator; (iii) a script to control the simulator’s behavior; and (iv) an embedded computer system for real-time implementation of the distracted driving detection algorithm and the alert system. The authors have adopted different CNN models including VGG-16, AlexNet, GoogleNet, and ResNet to classify driving activities. In the experiments, the distraction detection is performed by using the side camera to observe the driver’s hand and body movement. Ten subjects were asked to drive the car and conduct the ten activities. This system has the potential to be implemented in real cars, which can significantly reduce the traffic accidents caused by driving distractions.

## 3. Proposed Scheme

**Figure 1:**
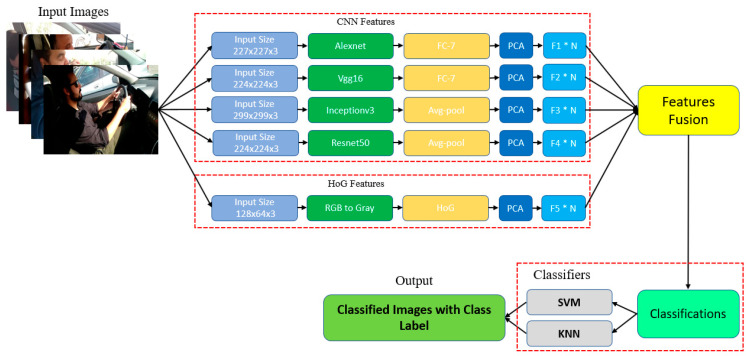
Architecture of HSDDD Hybrid Scheme for the Detection of Distracted Driving.

This section presents HSDDD, our proposed hybrid scheme for the Detection of Distracted Driving. The working of HSDDD is based on three-tiered architecture. The three tiers are named as Coordination tier, Concatenation tier, and Classification tier. Figure 1, illustrates the architecture and block diagram of our proposed scheme HSDDD.

First Tier is the Coordination tier in which feature extraction is performed. At the Coordination tier, features of distracted driving are extracted in parallel using four integrated pre-trained models including AlexNet, Inception V3, ResNet50, and VGG-16. These four models are trained on AUC dataset [13].

Second Tier is the Concatenation tier in which feature fusion is performed. At the Concatenation tier, features are concatenated, means the extracted features from the coordination tier are deeply fused with the features extracted from the HOG. This fusion process will generate an output which will become an input for the third tier called the Classification tier.

Third Tier is the Classification tier in which extracted features are classified using classification algorithm. At the Classification tier, the weights of the feature vectors are trained. After training, each distracted driving behavior is classified using different variants of KNN and SVM.

### 3.1. Coordination Tier

At the Coordination Tier, there is an integration of four pre-trained CNN models. These models take the input in the form of images of distracted drivers and extract features from these images in parallel so that the capability of deep learning can be enhanced. The four pre-trained models are AlexNet [30], ResNet50 [31], Inception V3 [32] and VGG-16 [33]. Before giving input to the coordination tier in the form of images from the training stage to the coordinated pre-trained models, preprocessing is necessary. Therefore, we perform preprocessing on the original images so that the input requirements of the four CNN models can be satisfied. In case of AlexNet, the image size should be 227 × 227 × 3. In case of ResNet50, the image size should be 224 × 224 × 3. In case of Inception V3, the image size should be 299 × 299 × 3 and for VGG-16 224 × 224 × 3 should be the image size.

#### 3.1.1. AlexNet Model

AlexNet model was proposed in 2012 by Alex Krizhevsky and his colleagues in the research paper [30]. It consists of eight layers with 62.3 million learnable parameters. AlexNet model has five convolutional layers. Moreover, there is an aggregate of a max pooling layer of 3 × 3 size with stride 2 and three fully connected (FC) layers. Each FC layer has 4096 neurons, except the final layer which has 1000 neurons. ReLU is applied as an activation function in all the layers but not in the output layer in order to accelerate by six times the training process speed. This model also has two dropout layers with the dropout rate set to be 0.5 to prevent the model form overfitting. At the last FC layer, Softmax is used as an activation function. The AlexNet network has the input of size (227 × 227) with the loss function used as cross entropy.

#### 3.1.2. ResNet50 Model

Before ResNet, the existing Deep Neural Network (DNN) was going deeper and deeper, resulting in a notorious vanishing gradient problem that make it hard to train the deep networks. This is because of the back-propagation of the gradient to earlier layers and resulted into infinitively small gradient due to the repeated multiplication. Thus, ResNet50 was introduced. ResNet50 short for Residual Network was proposed by Kaiming He, Xiangyu Zhang, Shaoqing Ren and Jian Sun in 2015 in their paper [31]. It is a specific type of neural network made up of Residual Blocks and proposed to solve the problem of training very deep networks. The skip connections (shortcut connection) are introduced in ResNet50 so that the vanishing gradient problem of deep neural networks can be resolved. This idea of skip connections provides gradient a shortcut path for flowing through the network. Resnet50 was proposed by Microsoft Research Asia in late 2015, with the purpose of solving the difficulty of training convolution neural network such as the increasing errors in training due to the neural network with increased number of layers. ResNet50, a variant of ResNet50, is a convolutional neural network that is 50 layers deep. Every two layers are connected forming a residual block. At stage 1, the architecture of this model consists of a convolutional kernel of (7 × 7) for extracting image features, Activation, Batch normalization and max pooling. There is an addition of the residual blocks of two types to the CNN from the stage 2 to stage 5. A CONV block is a residual block which is scaled has three convolutional kernels of (1 × 1) or (3 × 3). In the end, average pooling layer having (7 × 7) size kernel is utilized for the conversion of feature map in the output into feature vector which is 2048-dimensional.

#### 3.1.3. Inception V3 Model

In the progress of developing CNN classifiers, the Inception network was a significant achievement, concerning to deal with issues and complications that were creating because the information location had large amounts of variations. In a number of applications related to medical imaging, good performance has been achieved in classification tasks by leveraging transfer learning. Inception-v3 [32] is a CNN architecture from the Inception family with certain improvements such as increased accuracy and reduced computational complexity. Inception V3 has a combination of convolution kernel structure, which is sparse, and multiple subconvolution kernel structure, which is dense. It consists of (7 × 7) convolutions that are factorized. Label Smoothing is also performed by leveraging an auxiliary classifier so that label information can be easily proliferated to lower down the network. Furthermore, batch normalization for layers is also used in the side head. Factorizing Convolutions helps in reducing the number of connections/parameters without decreasing the network efficiency. In order to obtain different receptive fields, Convolutional kernels of different sizes, such as (1 × 1), (3 × 3) and (5 × 5), are utilized. When the input image is given, low latitude features of that image are extracted by the three convolution layers having 3 × 3 size kernels and one max pooling layer. For further extraction of features, two convolution layers having two kernels of size 3 × 3 and 1 × 1 and one max pooling layer are utilized. The three pooling layers are utilized so that features with high dimensions can be extracted from the images.

#### 3.1.4. VGG16 Model

VGG16 introduced by K. Simonyan and A. Zisserman from the University of Oxford [33] is a convolutional neural network model. The 16 in VGG16 refers to the fact that it has 16 layers that have weights. An image of size (224 × 224 × 3) is given to the input layer prediction results upon 1000 classes using a softmax is given through the output layer. This is an improved form of AlexNet in which large kernel sized filters are replaced by multiple (3 × 3) kernel-sized filters one after another. VGG16 architecture consists of (3 × 3) sized filter convolution layers with a stride 1. Through the whole architecture, it uses. same padding and (2 × 2) sized filter maxpool layer with stride 2 which is used consistently. In the end, it has 2 FC followed by a Softmax for output. VGG16 is large network, and it consists of 138 million parameters.

### 3.2. Histogram of Gradient (HOG) Features

HOG is short for Histogram of Oriented Gradients. It is a hand-crafted feature descriptor which is very popular for the detection of objects in the computer vision and image processing fields. It focuses on the structure or the shape of an object. It was initially proposed to be used for human detection [34]. The idea behind HOG is that appearance or shape of a local object can be represented by using gradient directions or distribution of edge. We use HOG to extract the edge features such as gradient and orientation of edges in the images. In the first step, the spatial gradients in horizontal and vertical directions are computed. The gradient magnitudes and angles are then computed by these two gradients. These orientations are calculated for the complete image in localized portions by breaking down the image into regions of smaller sizes called cells and in case of every region, the gradients and orientations are calculated. The size of the image should be (64 × 128) because for the extraction of features the image division will be done into (8 × 8) and (16 × 16) patches. In the second step, the gradient angle is used as a basis to vote each pixel gradient magnitude in a cell into different bins of orientation. In the third step, groups of the adjacent cells are made in the form of blocks and normalizing it in L2-norm. A descriptor is formed by concatenating the normalized block histograms in a detection window. Finally, a histogram for each of these regions is created separately by using the gradients and orientations of the pixel values.

### 3.3. Concatenation Tier

The second level of our proposed architecture is the Concatenation tier. At the Concatenation tier, the feature vectors are deeply fused. In our proposed scheme, there are two types of feature fusions. In the first type of feature fusion, the extracted features from the four CNN models are combined and then fused with the HOG extracted features. The output is then passed on to the Classification tier and the performance results are observed. In the second type of fusion, the extracted features of each of the four CNN models are fused (aggregated) separately with the HOG extracted features. The output of each separate fusion is passed on to the Classification tier, and the performance results are observed.

### 3.4. Classification Tier

The third level of our proposed architecture is the Classification Tier. At the Classification tier, training and testing is performed. First, in the training process, the training is performed by training the weights of the feature vectors. We have used variants of two classifiers, KNN, short for K-Nearest Neighbor, and SVM, short for Support Vector Machine. We have used six different SVM variants including Cubic SVM, Quadratic SVM, Linear SVM, Coarse Gaussian SVM, Medium Gaussian SVM and Fine Gaussian SVM. The four KNN variants include Cosine KNN, Coarse KNN, Medium KNN and Fine KNN. The evaluation of these classifiers is performed on the basis of some performance metrics. Experiments have shown that the best performance with highest accuracy is shown by Fine KNN and Cubic SVM classifiers. The experiments and the performance results are analyzed, presented and explained in detail in Section 4.

## 4. Experiments and Results

The major aim of this study is to develop a hybrid technique which is a combination of both handcrafted and deep learning that will work fine with the AUC distracted driving dataset. The proposed HSDDD methodology is used to extract deep features from four CNN models and fused them with extracted hand-crafted features. Then after applying feature selection, selected features are given to different variants of two classifiers, SVM and KNN, and then used to observe the system performance. The experiments for the evaluation of our proposed HSDDD methodology are performed on Intel(R) Core (TM) i7-3520M CPU @ 2.90 GHz with 8 GB RAM with windows 10 platform. Matlab2020a is used as the programming language.

### 4.1. Environment and Experimental Setup

**Figure 2:**
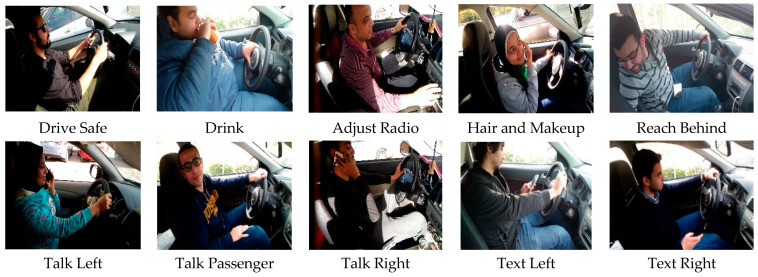
10 Classes of Driver Distractions.

The dataset of distracted driving behaviors comes from American University in Cairo (AUC) Distracted Driver Dataset, which is a publicly available dataset. The AUC dataset has 15 females and 29 males, a total of 44 individuals from seven countries: the USA, Canada, Germany, Morocco, Uganda, Egypt and Palestine. The collected input was in form of videos which were cut into individual images each of size (1080 × 1920). The videos were taken using different cars with different driving conditions, in different day timings, and the drivers were wearing different clothes. Figure 2 shows the distribution of 14,478 frames of the dataset over 10 classes. On the training set, we perform our experiments and the testing set images are unlabeled. Each image of a five-fold cross-validation scheme is utilized while performing training and testing which means that the dataset is divided into 80% random data, which is selected for the purpose of training, and for the purpose of testing, the remaining 20% data is selected.

**Figure 3:**
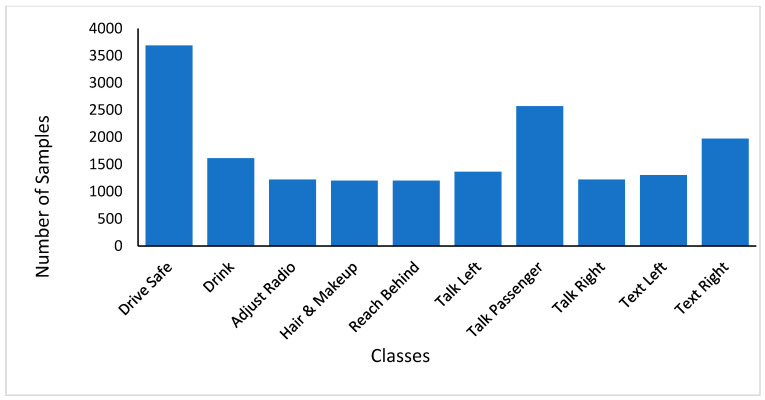
Total Number of Images in each Class.

The dataset is partitioned into ten classes of behavior of distracted drivers. These classes include safe driving, right-handed phone use, right-handed texting, left-handed phone use, left-handed texting, talking to passengers, glancing behind, operating the radio, hair and makeup, and drinking. We classify the ten classes of behaviors of distracted drivers, giving them labels as A–J. The number of distracted driving behavior samples in each class and total number of images in each class are shown in Figure 2 and Figure 3, respectively.

### 4.2. Experiments

The experiments are accomplished by selecting 100, 250 and 500 features from the FC layers of the four pre-trained CNN models. In different experiments, the performance of our proposed work is observed by taking a varying number of optimal features, and then classification is performed.

#### 4.2.1. Experiments Results with 100 Features

**Figure 4:**
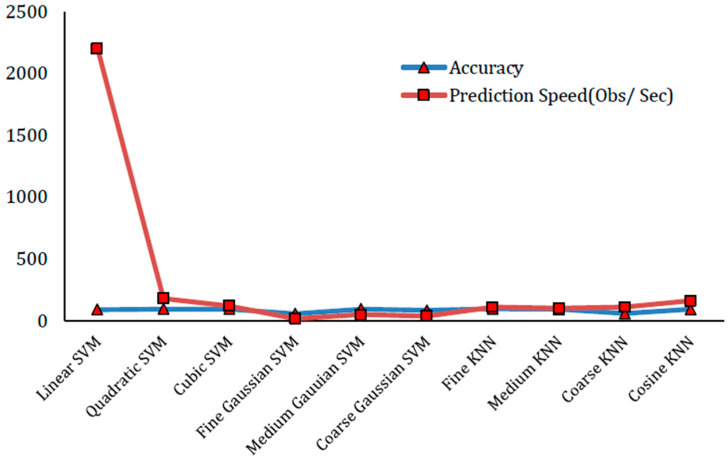
Prediction Speed (Obs/Sec) and Accuracy plot for best results with 100 features.

The first experiment involves the selection of 100 features. In this experiment, the feature matrix is of the size (17,308 × 100). On this feature matrix, we use five-fold cross-validation with ground truth class labels on AUC dataset [13]. The division of the dataset is in such a way that for training purposes, 80% random data is chosen, whereas for testing purposes, 20% remaining data is chosen. The prediction models which include the variants of SVM and KNN are used for automatic labeling by giving this feature matrix. In this test experiment, Fine KNN has provided the best result of 95.5% accuracy. Cubic SVM has shown the performance accuracy of 94.8% which is the second largest in terms of accuracy. Table 1 presents the experimental results and findings of this experiment with the selection of 100 features. The confusion matrix of Fine KNN classifier with 100 features is presented in Table 2. Similarly, Figure 4 shows a prediction speed (Obs/Sec) and accuracy plot in this experiment.

#### 4.2.2. Experiments Results with 250 Features

**Figure 5:**
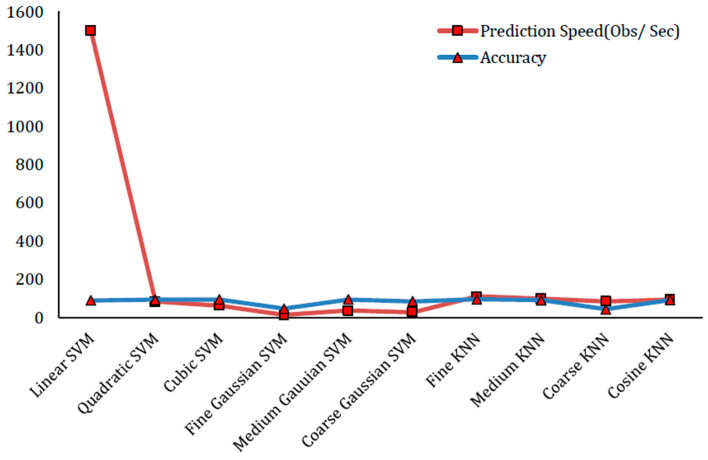
Prediction Speed (Obs/Sec) and Accuracy plot for best results with 250 features.

The second experiment involves the selection of 250 features. In this experiment, the feature matrix is of the size (17,308 × 250). On this feature matrix, we use five-fold cross-validation with ground truth class labels on AUC dataset. The division of the dataset is in such a way that for training purposes, 80% data is selected randomly whereas for testing purposes, 20% remaining data is chosen. The classification models for prediction include the variants of KNN and SVM which are used for automatic labeling by giving this feature matrix. In this test experiment, Fine KNN has provided the best result of 95.8% accuracy. Cubic SVM has shown the performance accuracy of 95.1% which is the second largest as far as accuracy is concerned. Table 3 presents the experimental results and findings of this experiment with the selection of 250 features. The confusion matrix of Fine KNN classifier with 250 features is presented in Table 4. Similarly, Figure 5 shows a prediction speed (Obs/Sec) and accuracy plot in this experiment.

#### 4.2.3. Experiments Results with 500 Features

**Figure 6:**
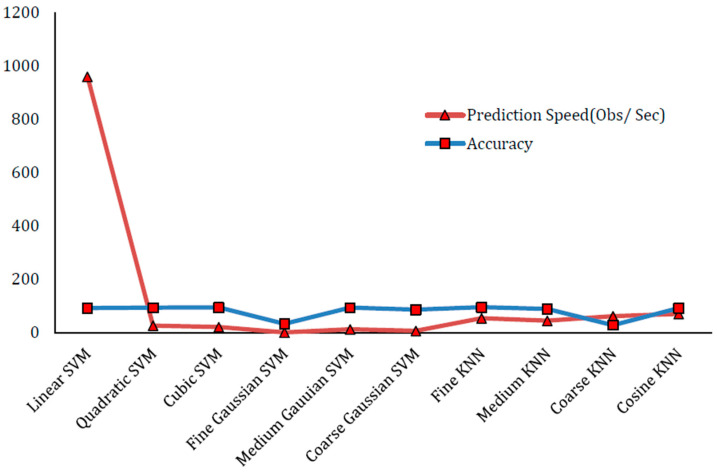
Prediction Speed (Obs/Sec) and Accuracy plot for best results with 500 features.

The third experiment involves the selection of 500 features. In this experiment, the feature matrix is of the size (17,308 × 500). On this feature matrix, we use five-fold cross-validation with ground truth class labels on AUC dataset. The division of the dataset is in such a way that for training purposes, 80% data is selected randomly whereas for testing purposes, 20% remaining data is chosen. The classification models for prediction include the variants of SVM and KNN which are used for automatic labelling by giving this feature matrix. In this test experiment, Fine KNN has provided the best result of 95.9% accuracy. Cubic SVM has shown the performance accuracy of 95.1% which is the second largest in terms of accuracy. Table 5 presents the experimental results and findings of this experiment with the selection of 500 features. The confusion matrix of FineKNN classifier with 500 features is presented in Table 6. Similarly, Figure 6 shows a prediction speed (Obs/Sec) and accuracy plot in this experiment.

For the performance evaluation of our proposed approach, all the experiments are performed on the same classifiers and accuracy (Ac), Precision (Pr), Recall and F1-measure are calculated for the best performance results of 100, 250 and 500 features as shown in Table 7.

### 4.3. Comparison with Existing Works

We selected several approaches [23,35,36,37,38,39,40] from the literature proposed for detecting distracted driving behaviors for the comparison with our proposed HSDDD framework. J. M. Mase et al. [23] have presented a driver distraction posture detection method in which first CNNs are leveraged for automatically learning the spatial posture features and then stacked Bidirectional Long Short-Term Memory (BiLSTM) Networks are used to capture the spectral-spatio features of the images by extracting the spectral features amongst the stacked feature maps from the pre-trained CNNs. K. Srinivasan et al. [35] have compared the performances of various CNN models using evaluation metrics and then best suited approach is determined based on these metrics. State Farm dataset is used for the experiments. M. D. Rifat et al. [36] have proposed a method in which the Alexnet architecture is modified, and then its features are aggregated with HOG features to detect distracted driving. Moslemi et al. [37] have presented a detection technique for the distracted drivers which is based on 3D CNN, and optical flow is utilized so that the detection accuracy can be improved. B. Baheti et al. [38] have proposed a CNN-based approach and have developed a new architecture, named mobileVGG, based on depth-wise separable convolutions and VGG16. A. Ezzouhri et al. [39] have used deep-learning-based segmentation for extracting the driver’s body parts, before performing the distraction detection and classification task. Using segmentation method, irrelevant objects are removed and driver’s critical body parts such as image regions that contribute to the driver’s distraction recognition are identified. The authors have also proposed a new annotated naturalistic driving image dataset for the study of driver distraction detection of more than 38K images. D. L. Nguyen et al. [40] have proposed a lightweight Convolutional Neural Network for a distracted driver warning system by combining standard convolution and Depth-wise Separable Convolution operation, and they have trained their network and evaluated on two datasets, AUC (the American University in Cairo) and StateFarm datasets. Table 8 demonstrates the comparative analysis of detection accuracies of some of the state-of-the-art approaches and our proposed approach on AUC dataset. It shows that all the existing techniques have used the accuracy metric for the evaluation of their techniques and most of them have not used other metrics like precision, recall and F1 score to validate and check the performance of their techniques.

### 4.4. Discussion

The manuscript is focused on the detection of distracted behavior of drivers. For this purpose, the AUC dataset is used. To increase the performance of the proposed approach, the benefits of handcrafted and deep learning are combined. The proposed method is an amalgam of both deep learning and handcrafted features. The deep features are used to extract the automatic features to represent the anomalous actions of the drivers. The HoG features are actually shape specialized handcrafted features. The combination of these two features makes the approach work effectively to detect the driver’s behavior with high accuracy. Moreover, we put our efforts with extensive experimentation performed on a number of tests on various deep learning as well as handcrafted feature extraction methods. In this manuscript, we only presented the most effective models that gave high accuracy.

Extensive experiments are performed. Our proposed model is trained on the AUC distracted driver dataset with ten classes. PCA is adopted for feature selection on both handcrafted and deep extracted features. To observe the performance of the proposed work, the classification is applied in different experiments with varying the number of optimal features. The results of experiments provided in Table 7 depict the performance of the proposed system in terms of accuracy (Ac), precision (Pr), recall and F1-measure. These results are obtained by varying the number of features (100, 250, 500) at the feature selection step.

According to our analysis of our experiments, the performance increases as the number of features increases, but after a certain level, the rate of increase of performance becomes very low. For instance, the accuracy difference between 100 features and 250 features is 0.3 percent. Similarly, the accuracy difference between 250 features and 500 features is 0.1 percent. Prediction speeds and accuracy plots of variants of KNN and SVM classifiers for 100, 250 and 500 features are shown in Figure 4, Figure 5 and Figure 6, respectively. The accuracy of SVM-based classifiers for 100 features is found in between 54.7% and 94.8%. The accuracy of SVM-based classifiers for 250 features is found in between 38.1% and 95.1%. The accuracy of SVM-based classifiers for 500 features is found in between 28.8% and 95.1%. The accuracy of KNN-based classifiers on the other hand for 100 features is found in between 56.9% and 95.5%. The accuracy of KNN-based classifiers for 250 features is found in between 37.6% and 95.8%. The accuracy of KNN-based classifiers for 500 features is found in between 27.2% to 95.9%. In all cases, the prediction speeds of KNN variants are relatively better as compared to SVM variants, except the linear SVM which has highest predictions speeds for 100, 250 and 500 features.

## 5. Conclusions

Driving behavior of a human driver has an important impact on safe driving, and thus it is found to be a significant factor that affects safety on roads. Driver distraction can cause serious injuries or may lead to loss of human lives, and thus, a human driver is required to be attentive and alert throughout the journey. In order to ensure a safe and secure trip, drivers have to make quick decisions and judgements based on cognition in such a road environment which is changing dynamically. To avoid severe road accidents and to improve road safety, it is necessary to monitor and predict early the human inattentive behavior including distracted behavior. The recent literature contains a number of research efforts to detect distracted human behavior by using deep-learning-based techniques. In this research article, we presented a Hybrid Scheme for the Detection of Distracted Driving called HSDDD based on a strategy of aggregating handcrafted and deep CNN features. HSDDD is based on three-tiered architecture. The three tiers are named Coordination tier, Concatenation tier and Classification tier. We first obtained HOG features by using handcrafted algorithms, and then at the Coordination tier, we leveraged four deep CNN models including AlexNet, Inception V3, Resnet50 and VGG-16 for extracting DCNN features. DCNN extracted features were fused with HOG extracted features at the Concatenation tier. Then PCA was used as feature selection technique for removing the redundant and irrelevant information as well as improving the classification performance. After feature fusion and feature selection, the selected features were passed to two types of classifiers, SVM and KNN, at the level of the Classification tier, and the performances of their variants were observed. The performance results concluded from the experiments and comparison of our proposed methodology with the state-of-the-art approaches have shown that combination of handcrafted features and deep features can provide a better solution for the detection of distracted driving. Although we achieve notable results, still there is a limitation on more accuracy. This domain can further be explored on various state-of-the-art approaches such as quantum computing and brain-like computing.

## Figures and Tables

**Table 1:** Accuracies of Classifiers for Experiments with 100 Features.

Classifier/Features	Alexnet + HOG	Inception + HOG	Resnet-50 + HOG	Vgg-16 + HOG	All
Linear SVM	83.4	81.6	84.5	82.7	88.9
Quadratic SVM	92.8	92.1	93	92.8	93.7
Cubic SVM	**94.2**	**93.9**	**94.4**	**94.3**	**94.8**
Fine Gaussian SVM	67.3	54.7	65.1	62.7	55.5
Medium Gauuian SVM	93.4	92.8	93.5	93.6	94.1
Coarse Gaussian SVM	77.3	75.4	78	76.5	83.3
Fine KNN	**95.1**	**94.8**	**95.1**	**95.1**	**95.5**
Medium KNN	90.3	90.3	90.6	90.6	91.2
Coarse KNN	58	59.7	59.1	57.8	56.9
Cosine KNN	90.7	90.4	90.8	90.9	91.5

**Table 2:** Confusion Matrix of FineKNN Classifier with 100 Features.

**Drive Safe**	**3523**	18	14	24	22	11	50	-	-	24
**Drink**	32	**1550**	9	-	4	-	12	-	3	2
**Adjust Radio**	45	9	**1145**	1	2	8	-	-	4	6
**Hair and Makeup**	57	6	-	**1122**	5	1	11	-	-	-
**Reach Behind**	61	7	1	9	**1073**	-	8	-	-	-
**Talk Left**	7	-	4	-	-	**1308**	-	-	40	2
**Talk Passenger**	70	8	-	6	4	-	**2478**	-	1	3
**Talk Right**	1	2	-	-	-	1	1	**1164**	11	43
**Text Left**	2	2	1	-	-	23	-	13	**1244**	16
**Text Right**	23	1	3	-	-	1	1	19	9	**1917**
	Drive Safe	Drink	Adjust Radio	Hair and Makeup	Reach Behind	Talk Left	Talk Passenger	Talk Right	Text Left	Text Right

**Table 3:** Accuracies of Classifiers for Experiments with 250 Features.

Classifier/Features	Alexnet + HOG	Inception + HOG	Resnet-50 + HOG	Vgg-16 + HOG	All
Linear SVM	89.6	88.8	89.9	89.4	92.3
Quadratic SVM	93.5	92.9	93.8	93.6	94.4
Cubic SVM	**94.6**	**94.1**	**94.8**	**94.6**	**95.1**
Fine Gaussian SVM	51.9	38.1	46.4	48.4	52.1
Medium Gaussian SVM	94.2	93.7	94.3	94.1	94.6
Coarse Gaussian SVM	84.4	82.9	84.9	84.5	87.3
Fine KNN	**95.5**	**95.3**	**95.8**	**95.7**	**95.7**
Medium KNN	90.5	90.3	91.2	90.9	91
Coarse KNN	42	40.4	44.1	42.4	37.6
Cosine KNN	91.5	91.6	91.8	91.8	92.1

**Table 4:** Confusion Matrix of FineKNN Classifier with 250 Features.

**Drive Safe**	**3543**	19	17	24	15	10	38	-	-	20
**Drink**	29	**1549**	10	2	4	-	10	2	3	3
**Adjust Radio**	40	8	**1155**	1	1	8	1	-	4	2
**Hair and Makeup**	48	7	-	**1133**	4	-	10	-	-	-
**Reach Behind**	53	15	1	4	**1076**	-	9	-	-	1
**Talk Left**	5	-	3	-	-	**1311**	1	-	40	1
**Talk Passenger**	66	6	-	6	7	-	**2483**	-	-	2
**Talk Right**	1	2	-	-	-	-	-	**1171**	9	40
**Text Left**	3	4	1	-	-	23	-	14	**1245**	11
**Text Right**	26	2	2	-	-	-	1	14	11	**1918**
	Drive Safe	Drink	Adjust Radio	Hair and Makeup	Reach Behind	Talk Left	Talk Passenger	Talk Right	Text Left	Text Right

**Table 5:** Accuracies of Classifiers for Experiments with 500 Features.

Classifier/Features	Alexnet + HOG	Inception + HOG	Resnet-50 + HOG	Vgg-16 + HOG	All
Linear SVM	92	90.8	92.3	91.9	93.4
Quadratic SVM	94.1	93.3	94.3	94	94.8
Cubic SVM	**94.7**	**94**	**94.8**	**94.5**	**95.1**
Fine Gaussian SVM	36.6	28.8	32.8	35.8	93.4
Medium Gauuian SVM	94.1	93.3	94.2	94.1	94.3
Coarse Gaussian SVM	86.4	83.9	86.1	85.7	88.4
Fine KNN	**95.5**	**95.2**	**95.9**	**95.6**	**95.8**
Medium KNN	88.6	86	88.8	88	87.9
Coarse KNN	27.4	27.2	28.6	27.8	27.2
Cosine KNN	92.2	92.3	92.5	92.4	92.6

**Table 6:** Confusion Matrix of FineKNN Classifier with 500 Features.

**Drive Safe**	**3554**	15	12	21	16	7	42	-	-	19
**Drink**	35	**1548**	8	-	5	-	11	2	1	2
**Adjust Radio**	46	7	**1152**	1	-	8	1	-	4	1
**Hair and Makeup**	59	5	1	**1125**	3	-	9	-	-	-
**Reach Behind**	64	11	1	4	**1068**	-	10	-	-	1
**Talk Left**	5	1	3	-	-	**1312**	-	-	39	1
**Talk Passenger**	62	8	-	4	4	-	**2492**	-	-	-
**Talk Right**	2	-	-	-	-	-	-	**1174**	9	38
**Text Left**	2	3	-	-	-	21	-	13	**1251**	11
**Text Right**	22	2	2	-	-	-	1	17	9	**1921**
	Drive Safe	Drink	Adjust Radio	Hair and Makeup	Reach Behind	Talk Left	Talk Passenger	Talk Right	Text Left	Text Right

**Table 7:** Comparative analysis of various performance under different selected features.

No of Selected Features	Best Accuracy	Precision	Recall	F1 Score
100	95.5%	0.952	0.961	0.956
250	95.8%	0.924	0.941	0.932
500	95.9%	0.954	0.963	0.960

**Table 8:** Comparative analysis on AUC dataset.

Approaches	Accuracy	Precision	Recall	F1 Score
Our Approach	95.90%	95.4%	96.3%	96%
[35]	-	92.53%	94.85%	94.18%
[36]	93.19%	-	-	-
[37]	73%	75.3%	77.1%	-
[23]	92.70%	92.8%	92.7%	92.8%
[38]	95.24%	-	95.19%	-
[39]	95.77%	-	-	-
[40]	95.36%	-	-	-

## Data Availability

Data is available upon request.
